# 

**Published:** 1895-04-13

**Authors:** 


SATURDAY, APRIL 13TH. 1895.

With Index for Vol. XVII

WITH EXTRA NURSING SUPPLEMENT.

[Registered at the General Post Office as a Newspaper for transmission in the United Kingdom and Abroad.]

PRICE TWOPENCE.

Per Annum, 10s. 6d., post free.

Monthly Parts, 6d.; post free, 8d.

Ditto per Annum, 8s. 6d., post free.

